# Calsequestrin: a well-known but curious protein in skeletal muscle

**DOI:** 10.1038/s12276-020-00535-1

**Published:** 2020-12-07

**Authors:** Jin Seok Woo, Seung Yeon Jeong, Ji Hee Park, Jun Hee Choi, Eun Hui Lee

**Affiliations:** 1 Department of Physiology, David Geffen School of Medicine, UCLA, Los Angeles, CA 10833 USA; 2 Department of Physiology, College of Medicine, The Catholic University of Korea, Seoul, 06591 Korea; 3 Department of Biomedicine & Health Sciences, Graduate School, The Catholic University of Korea, Seoul, 06591 Korea

**Keywords:** Physiology, Calcium and vitamin D, Diseases

## Abstract

Calsequestrin (CASQ) was discovered in rabbit skeletal muscle tissues in 1971 and has been considered simply a passive Ca^2+^-buffering protein in the sarcoplasmic reticulum (SR) that provides Ca^2+^ ions for various Ca^2+^ signals. For the past three decades, physiologists, biochemists, and structural biologists have examined the roles of the skeletal muscle type of CASQ (CASQ1) in skeletal muscle and revealed that CASQ1 has various important functions as (1) a major Ca^2+^-buffering protein to maintain the SR with a suitable amount of Ca^2+^ at each moment, (2) a dynamic Ca^2+^ sensor in the SR that regulates Ca^2+^ release from the SR to the cytosol, (3) a structural regulator for the proper formation of terminal cisternae, (4) a reverse-directional regulator of extracellular Ca^2+^ entries, and (5) a cause of human skeletal muscle diseases. This review is focused on understanding these functions of CASQ1 in the physiological or pathophysiological status of skeletal muscle.

## Muscle function: Multiple roles for a muscle modulator

Although previously considered merely a passive regulator of calcium levels, the protein calsequestrin is now known to perform a range of physiological activities essential to skeletal muscle function. The process of muscle contraction depends on the release of calcium ions from an intracellular structure called the sarcoplasmic reticulum (SR). Calsequestrin was originally identified as a “buffering” factor that maintains adequate calcium reserves in the SR, but Eun Hui Lee and colleagues at the Catholic University of Korea review diverse functions that have since been ascribed to this protein. For example, calsequestrin also helps reinforce the structure of the SR, and actively regulates the flux of calcium ions into muscle cells. Perturbations in calsequestrin function also appear to contribute to a number of muscular disorders, including a potential link to Duchenne muscular dystrophy.

## Introduction

Generally, the Ca^2+^ supply in intracellular Ca^2+^ signals consists of two Ca^2+^ pools: internal Ca^2+^ (in the endoplasmic or sarcoplasmic reticulum (ER or SR)) and extracellular Ca^2+^. To sustain body postures and perform body movements, skeletal muscle contracts or relaxes, and the main key elements governing skeletal muscle contraction and relaxation are well understood^1,2^. During contraction and relaxation, the movements of contractile proteins in skeletal muscle cells (myotubes in culture conditions and fibers from skeletal muscle tissue) are mainly dependent on the cytosolic level of Ca^2+^ ions that are released from the SR. Dihydropyridine receptor (DHPR, a voltage-gated Ca^2+^ channel (i.e., Cav1.1.) on the transverse (t)-tubule membrane) senses the depolarization of the t-tubule membrane (i.e., action potentials) in response to acetylcholine that is released from motor neurons. T-tubules are invaginations of the plasma membrane and have a transverse orientation with respect to the main axis of myofibrils. The conformational changes in DHPR due to the action potential subsequently activate ryanodine receptor type 1 (RyR1, an internal Ca^2+^ release channel on the SR membrane) via physical interactions (not all RyRs but every other RyR). The interaction allows the release of Ca^2+^ from the SR to the cytosol through RyR1. The released Ca^2+^ from the SR turns on contractile proteins and evokes skeletal muscle contraction. In short, a transient elevation in cytosolic Ca^2+^ levels due to Ca^2+^ release from the SR couples the action potential and muscle contraction (so-called “excitation–contraction (EC)” coupling). EC coupling is defined as an “orthograde signal” from DHPR in the t-tubule membrane to RyR1 in the SR membrane (to distinguish it from the retrograde signal from RyR1 to DHPR or the reverse-directional signals from proteins in the SR or on the SR membrane to proteins or events in the t-tubule/plasma membrane). Three different genes on chromosomes 19, 1, and 15 encode three distinct isoforms of RyRs (RyR1, RyR2, and RyR3). RyR1 is predominant in skeletal muscle, whereas RyR3 is expressed at much lower amounts than RyR1 in skeletal muscle. In addition to the physical interaction of DHPR and RyR1, the Ca^2+^ that is released from SR binds and activates nearby RyR1, which does not interact physically with DHPR. This is called Ca^2+^-induced Ca^2+^ release, which contributes to rapid intracellular Ca^2+^ release from the SR and to the amplification of Ca^2+^-mediated signals^3^.

Cytosolic [Ca^2+^] at rest (nM range) rises to the μM range during skeletal muscle contraction^4^. To establish the Ca^2+^ increase, in addition to the Ca^2+^ from the SR, extracellular Ca^2+^ entry via Ca^2+^ channels in the t-tubule membrane, such as Orai1 or transient receptor potential canonical type (TRPC), also participates in the cytosolic Ca^2+^ increase, especially for skeletal muscle contraction during tetanic stimulation or fatigue^5–7^. The relaxation of skeletal muscle involves the reuptake of Ca^2+^ from the cytosol to the SR to reset the resting cytosolic Ca^2+^ level and replenish SR Ca^2+^. Sarcoplasmic/endoplasmic reticulum Ca^2+^-ATPase (SERCA, a pump in the ER/SR membrane) is mainly responsible for Ca^2+^ reuptake, and SERCA1a is the major isoform in adult skeletal muscle^1,8^. In addition to SERCA, the Na^+^/Ca^2+^ exchanger (NCX) in the plasma membrane, mNCX in the mitochondrial membrane or Ca^2+^-ATPase in the plasma membrane (called PMCA) also participate in removing Ca^2+^ from the cytosol under certain conditions, such as a Ca^2+^ increase involving store-operated Ca^2+^ entry (SOCE)^9–11^. The efficient arrangement of EC coupling-mediating proteins mentioned above along with their regulatory proteins in the triad junction (a specialized membrane structure in skeletal muscle cells, see “SR and triad junction” in this review) is crucial for their proper functions and intermolecular interactions^12–14^. Therefore, for the successful cycling of the rise and fall in cytosolic Ca^2+^ level during repetitive skeletal muscle contraction and relaxation during a lifetime, the spatiotemporal distributions of intracellular Ca^2+^ ions, timely expression of EC coupling-mediating proteins with their regulatory proteins at different levels, and formation of triad junctions should be coordinately prepared with pinpoint accuracy.

## SR and triad junction

SR, a skeletal muscle form of ER, is a very dynamic intracellular organelle involved in the reserve, release, and reuptake of Ca^2+^^1,15–18^. SR is morphologically very specialized and distinct from the ER^16,19,20^, which is required for efficient and immediate Ca^2+^ release from the SR to the cytosol via RyR1. The SR is divided into two functionally distinct but continuous portions: the longitudinal SR (LSR) and the terminal cisternae. LSR is responsible for the largest part of the SR and is the site of Ca^2+^ reuptake from the cytosol via SERCA^21,22^. Terminal cisternae can be subdivided into junction-facing SR (JSR) and the remaining part^16,23^. JSR (also roughly called terminal cisternae in many other articles) is located in close proximity to t-tubules. The membrane structure that comprises two JSRs and a t-tubule between the two JSRs, such as a sandwich, is called the “triad junction”. Close arrangements of the JSR and t-tubule membranes in the triad junction allow physical interaction between DHPR in the t-tubule membrane and RyR1 in the JSR membrane. Therefore, the triad junction is a functional structure for the cytosolic Ca^2+^ increase during EC coupling.

The formation of the triad junction is primarily mediated by junctophilins (JPHs). Each JPH contains eight repeats of hydrophobic motifs (called the membrane occupation and recognition nexus (MORN)) and a C-terminal transmembrane domain spanning the JSR membrane^12^. MORN motifs confer the ability to attach to phospholipids in the t-tubule membrane, which causes JPHs to serve as bridges between the t-tubule and SR membranes and subsequently allow close and parallel positioning of the t-tubule and SR membranes in the triad junction^12,13^. JPH1 and JPH2 are expressed in adult skeletal muscle at different frequencies: JPH1 is found throughout the triad junction when RyR1 is present, and JPH2 is present at a reduced frequency compared to JPH1^24^. In addition to their role as a structural bridge in the triad junction, JPHs also play functional roles by interacting with SR proteins and regulating Ca^2+^ movements in skeletal muscle. JPH1 can interact with RyR1^25^. Both JPH1 and JPH2 can interact with DHPR^26^. JPH2, but not JPH1, can interact with TRPC3^27,28^. JPH1 knockout mice show a reduction in the number of triad junctions and structural abnormalities in the SR, such as a vacuolated LSR and swollen triad^29^. Skeletal muscle fibers from JPH1 knockout mice induce a reduction in the expression of TRPC3, Orai1, and stromal interaction molecule 1 (STIM1), as well as reductions in Orai1-mediated SOCE, cytosolic Ca^2+^ levels, and Ca^2+^ amounts in the SR^30^. For details on SOCE, see “A reverse-directional signal from CASQ1 to SOCE” in this review. Knockdown of both JPH1 and JPH2 in C2C12 myotubes (a skeletal muscle cell line) leads to deformation of the junctional membrane complex (corresponding to the triad junction of skeletal muscle fibers), reductions in SOCE and Ca^2+^ amounts in the SR, and elevation of the cytosolic Ca^2+^ level^31^. S165F, a JPH2 mutant that is found in patients with hypertrophic cardiomyopathy, induces hypertrophic skeletal myotubes along with a reduction in RyR1 activity during EC coupling due to a defect in protein kinase C (PKC)-mediated phosphorylation at Ser165 and the subsequent absence of the interaction between JPH2 and TRPC3^32^. Another JPH2 mutant that is found in other patients with hypertrophic cardiomyopathy, Y141H, also shows hypertrophic skeletal myotubes due to an abnormal junctional membrane complex and increased Orai1-mediated SOCE^13^. An interaction between TRPC3 and RyR1 has been reported^28,33^. TRPC3 knockdown in skeletal myotubes results in decreased RyR1 activity and increased expression of JPH1 along with other proteins (TRPC1, CASQ1, and triadin)^34^. These various reports suggest that all these proteins are physically and functionally related to one another during Ca^2+^ movements in skeletal muscle.

Mitsugumin 29 (MG29) also participates in the formation of triad junctions during myogenesis or the terminal differentiation of myoblasts (proliferative forms of satellite cells or immature skeletal muscle cells) to myotubes (mature skeletal muscle cells)^14^. MG29 knockout mice exhibit ultrastructural abnormalities in the triad junction, such as swollen and irregularly directed t-tubules, irregular SR structures, and partial triad junction misformation, along with functional abnormalities such as reduced twitch tension under Ca^2+^-free conditions. For more details on the formation of triad junctions and the equipment of Ca^2+^-handling proteins such as DHPRs, RyRs, SERCAs, and CASQs during the development of skeletal muscle, please refer to other reviews and research articles^35,36^.

In addition to the SR, mitochondria are the second most important intracellular Ca^2+^ store in skeletal muscle, and they are closely localized in triad junctions to functionally communicate with the SR^37–40^, which is a very interesting field for further detailed investigation. For details on the relationship between mitochondria and the SR in regulating various Ca^2+^-mediated cellular events, please refer to another review articles^41–43^.

## Properties of CASQ1 and isoform transition of CASQs during development

CASQ1 is a protein that has been known since its discovery 50 years ago in rabbit skeletal muscle (1971)^15,44^. The cDNA of CASQ1 was cloned from fast-twitch skeletal muscle from rabbits^45^, and the cDNA of the cardiac isoform of CASQ (CASQ2) was cloned from the heart and slow-twitch skeletal muscle from canines^46^. CASQ1 is the major isoform in adult fast-twitch skeletal muscles, while CASQ2 is the only isoform in cardiac muscle and a minor isoform in adult slow-twitch skeletal muscle^46,47^. Smooth muscle expresses both isoforms^15,44,48^.

The *CASQ1* gene is located on human chromosome 1q21^49^. CASQ1 presents a high degree of homology to CASQ1 from other species or to CASQ2 in amino acid sequences^50^: for example, 98% between rabbit and human CASQ1s 86% between dog and human CASQ2s, and 83% between rabbit CASQ1 and dog CASQ2. The crystal structure of CASQ1 is nearly superimposable on that of CASQ2^51–53^. Despite these high homologies, CASQ1 differs from CASQ2 in its C-terminus (a highly extended acidic C-terminus in CASQ2)^50^. The role of CASQ1 in regulating RyR1 activity differs from that of CASQ2; for example, in single-channel studies in planar lipid bilayers, CASQ2 monomers and/or dimers activate both RyR1 and RyR2, while CASQ1 polymers inhibit RyR1 activity^54^. The C-termini of CASQs contains one (CASQ1) or two glycosylation sites (CASQ2), as well as one (CASQ1) or three phosphorylation sites (CASQ2) for casein kinase II (CKII)^55,56^. The phosphorylation of CASQ1 is discussed further in a later section of this review.

CASQ1 and CASQ2 are heavily enriched in the SR, and they are tightly associated with the membrane of JSRs but not LSRs (in the case of rabbit CASQ1, approximately 70% after a purification process)^15,18,44,51,54,57–59^. CASQs are not evenly distributed in the SR but are concentrated in the vicinity of JSR-bearing RyR1 arrays^16,60^. For example, CASQ1 is located within 100 nm around RyR1 when it is not anchored to RyR1 or within 20–50 nm when it is anchored to RyR1. Electron microscopy (EM) studies have revealed that CASQ1 (as a form of homopolymer) shows a thin strand-like appearance in the SR and confirms the association of CASQ1 with the SR membrane^61,62^.

During embryonic development, maturation, or adulthood, the degree of expression of CASQ1 or CASQ2 in fast- and slow-twitch skeletal muscle is dependent on the skeletal muscle type. In a study using a mixture of rabbit fast- and slow-twitch skeletal muscles, both CASQ1 and CASQ2 were present from the embryonic stage, and fast-twitch skeletal muscle displayed more CASQs than slow-twitch skeletal muscle^63^. The expression of CASQ1 and CASQ2 steeply increased before birth and reached adult values at 4 days after birth. Denervation of postnatal fast- and slow-twitch skeletal muscles decreased the expression levels of CASQ1 and CASQ2 to their levels at the embryonic stage. In addition, during the postnatal development of rabbit skeletal muscle (i.e., during postnatal myogenesis), protein–protein interactions that are involved in EC coupling become more complex and mature for the proper physiological functions of skeletal muscle. The expression of CASQ1 and CASQ2 in the mixture of rabbit fast- and slow-twitch skeletal muscle continued to increase until day 41 and resembled that in adult skeletal muscle^36^.

Both CASQ1 and CASQ2 are expressed in neonatal rabbit fast-twitch skeletal muscle, and a transition of CASQ isoforms occurs after birth^64^. The expression of CASQ1 increased up to 1 month, and it reached adult values at approximately 2 months of age. The expression of CASQ2 completely disappeared between 2 and 4 weeks postnatally (i.e., during the maturation of fast-twitch skeletal muscle, downregulation of CASQ2 expression and gradual replacement of CASQ2 with CASQ1). The amount of CASQ1 in the rat fast-twitch extensor digitorum longus (EDL) skeletal muscle fibers was 3.5 times greater than that in slow-twitch soleus skeletal muscle fibers^65^, in accordance with the higher amount of the released Ca^2+^ in fast-twitch than in slow-twitch skeletal muscles. Both isoforms are present in rabbit slow-twitch skeletal muscle until adulthood, and CASQ2 accounts for approximately 25% of the total CASQs^66^.

## CASQ1 is a major high-capacity Ca^2+^-buffering protein and a Ca^2+^-sensor in the SR

**Fig. 1:**
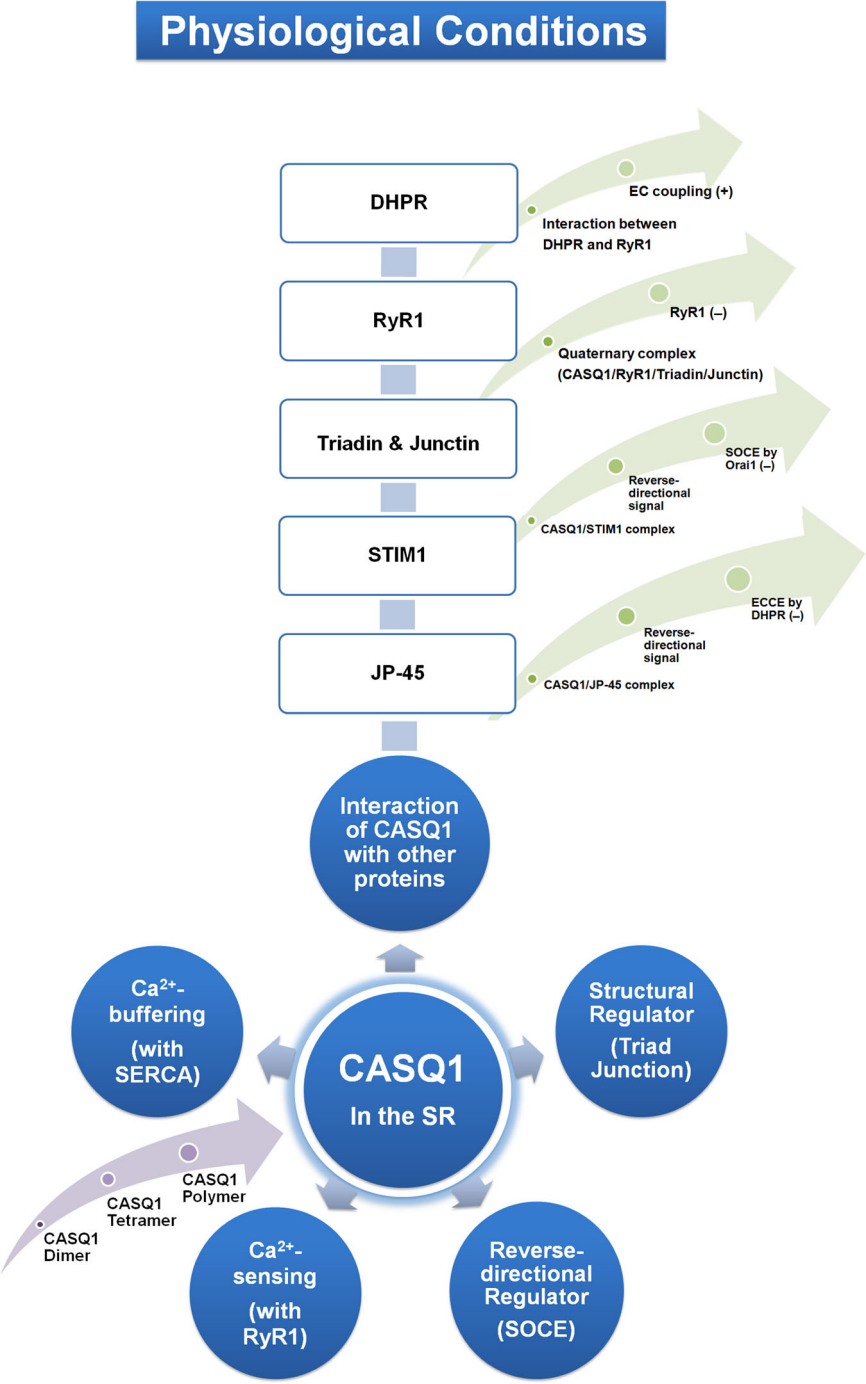
Summary of the physiological roles of CASQ1 in skeletal muscle. The physiological roles of CASQ1 in skeletal muscle are summarized in circles (colored blue). Within the circles, corresponding proteins, mechanisms, or events are presented in parentheses. The light purple arrow (in the lower left) indicates the process of CASQ1 polymerization. Proteins that interact with CASQ1 are listed in boxes. Greenish arrows indicate the protein complexes of CASQ1 with CASQ1-interacting proteins along with the effects of the complexes on the functions or protein activities of skeletal muscle. (+), positive or upregulation; (−), negative or downregulation. CASQ1 calsequestrin 1, DHPR dihydropyridine receptor, RyR1 ryanodine receptor 1, STIM1 stromal interaction molecule 1, JP-45 junctional protein 45, SERCA sarcoplasmic/endoplasmic reticulum Ca^2+^-ATPase, EC coupling excitation-contraction coupling, SOCE store-operated Ca^2+^ entry, ECCE Ca^2+^ entry through the DHPR during skeletal EC coupling.

The first role of CASQ1 in skeletal muscle is to buffer Ca^2+^ in the SR with a low-affinity and high-capacity Ca^2+^-binding ability (40–50 moles or maximally ~80 moles of Ca^2+^/1 mole of CASQ1) to prepare rapidly releasable Ca^2+^ during EC coupling (Fig. 1)^15,67,68^. Ca^2+^-binding sites in CASQ1 are very different from those in other Ca^2+^-binding proteins (i.e., they do not consist of EF-hands). The role of CASQ1 as a high-capacity Ca^2+^-buffering protein is accomplished by two factors^51^. First, CASQ1 is an extremely acidic protein. Two-thirds of the C-terminus of CASQ1 is negatively charged, and it serves as the Ca^2+^-binding site^51^. Second, CASQ1 self-polymerizes to prepare high-capacity Ca^2+^-binding at high [Ca^2+^] in the SR (i.e., Ca^2+^-dependent homopolymerization of CASQ1 near RyR1, further discussed in the next section of this review)^53,69^. The polymerization of CASQ1 in skinned fibers from rat skeletal muscle theoretically increases the total [Ca^2+^]_SR_ up to 20 mM, and simultaneously, it maintains a free [Ca^2+^]_SR_ at approximately 1 mM^50,70^, which is important for easier and more efficient Ca^2+^ reuptake into the SR by SERCA1a during relaxation and for the storage of high [Ca^2+^] in the SR without harmful osmotic effects. For example, frog skeletal muscle fibers with CASQ1 bear 23 times more Ca^2+^ than those without CASQ1^71^.

The second role of CASQ1 in skeletal muscle is as a Ca^2+^ sensor in the SR. CASQ1 senses [Ca^2+^] in the SR and modulates Ca^2+^ release from the SR to the cytosol via RyR1 in a conformation (i.e., polymerization)-dependent manner (Fig. 1)^67,68,72,73^. Lowering the [Ca^2+^]_SR_ from 1 mM to 100 µM results in conformational changes in CASQ1 (the dissociation of 65–75% of CASQ1 from the JSR membrane). Under this condition, CASQ1 is depolymerized and no longer inhibits RyR1 activity. CASQ1 also protects the SR from deep Ca^2+^ depletion, which causes massive migration of CASQ1 away from the JSR membrane and remodeling of the SR^74,75^. Under long-lasting membrane depolarization, such as high-frequency stimuli or early stages of muscle fatigue, Ca^2+^ in the SR is not fully but partly depleted due to the inactivation of RyR1 at a certain moment by limiting the further depolymerization of CASQ1, which prevents excess Ca^2+^ release from the SR and catastrophic changes in the SR structure. Therefore, it can also be said that by conformational changes (polymerization or depolymerization) in a Ca^2+^-dependent manner, CASQ1 senses the degree of Ca^2+^ depletion from the SR and regulates RyR1 activity. In addition, the regulation of RyR1 activity by CASQ1 requires interactions with other proteins (discussed in ‘Regulation of RyR1 activity by a quaternary complex of CASQ1/RyR1/triadin/junctin’ in this review).

## Three-dimensional (3D) structure and polymerization of CASQ1

The 3D structure of CASQ1 was determined at a resolution of 2.4 Å^53^. The CASQ1 monomer has three almost identical domains (I–III), each with a topology similar to that of thioredoxin from *Escherichia coli* (i.e., three thioredoxin-like folds occur in CASQ1). Each domain forms a disk-like shape with a fold of *α*/*β* structures (a five-stranded β-sheet in the core flanked by four α-helices (two on each side of the β-sheets). The thioredoxin-like fold occurs at ~10 μM [Ca^2+^]. The roles of the thioredoxin-like domain in CASQ1 have not been studied. Considering that the thioredoxin system participates in the regulation of metabolism, the antioxidant system, cell signaling pathways, and thioredoxin-dependent chaperone actions^76^, the presence of the three thioredoxin-like domains in CASQ1 suggests that CASQ1 could be directly or indirectly involved in the thioredoxin system.

CASQ1 can exist either as monomers or homopolymers (a wide range of high molecular masses), and polymeric CASQ1 allows various binding to other proteins via the same region, such as the asp-rich/CAS (consecutive aspartate stretch) region. The polymerization of CASQ1 depends on [Ca^2+^]_SR_^50,53,73,77,78^. At 10 μM [Ca^2+^]_SR_, CASQ1 exists as a monomer and dissociates from the JSR membrane, which eliminates the inhibitory effect of CASQ1 on RyR1. Two types of dimers facilitate the polymerization of CASQ1. The first type of dimer occurs via a back-to-back interaction. Salt bridges between Glu215-Lys86, Glu215-Lys24, and Glu169-Lys85 from domains I and II stabilize the back-to-back interaction. Two salt bridges between Lys85-Glu223 and between Lys86-Glu215 mediate an interaction between the dihydrobasic sequence (found in several Ca^2+^-binding proteins^79^) and a binding site for trifluroperazine (an antipsychotic drug), which also contributes to the back-to-back interaction. In the back-to-back interaction, the C-terminal asp-rich/CAS region from each monomer forms a negatively charged pocket within the dimer, and the negative pocket represents a major Ca^2+^-binding motif in CASQ1^80^. At low [Ca^2+^], the asp-rich/CAS region can accommodate 6–8 Ca^2+^ ions, which neutralizes the acidic property of the asp-rich/CAS region and allows back-to-back stacking. The remaining regions of CASQ1 contain additional Ca^2+^-binding sites that can progressively support more Ca^2+^-binding as [Ca^2+^] increases^80^. The back-to-back interaction during the polymerization of CASQ1 occurs at 10 μM to 1 mM [Ca^2+^]_SR_.

The second type of dimer occurs via a front-to-front interaction. Tetramerization of CASQ1 occurs via a front-to-front interaction between two dimers (via the N-terminus) that are formed by the back-to-back interaction between two monomers (via the C-terminus) when [Ca^2+^] is further increased. The front-to-front interaction occurs with the insertion of the N-terminal part of one monomer of the dimer into a hydrophobic cleft in the adjacent monomer of another dimer. This process again generates a negatively charged pocket that supports Ca^2+^-binding. A salt bridge between Glu55–Lys49 contributes to the front-to-front interaction and helps to stabilize CASQ1 polymers. Further polymerization of the CASQ1 tetramers finally forms thin strands (also called ribbon-like linear polymers) that generate more negative pockets, which confer a higher Ca^2+^-buffering capacity and the ability to bury hydrophobic side chains of amino acids (i.e., losing hydrophobicity) on CASQ1. The highly extended structure of polymeric CASQ1sbecomes more compact by the binding of more Ca^2+^^81^. Polymeric CASQ1 is stably anchored to the JSR membrane by binding to other proteins (i.e., triadin and junctin along with RyR1) at ~1 mM [Ca^2+^]_SR_ and can maximally inhibit RyR1 activity^50^. The polymerization of CASQ1 is inhibited by increasing [K^+^]. From these reports, both terms of Ca^2+^-dependent polymerization of CASQ1 or polymerization-dependent Ca^2+^-binding of CASQ1 are correct. The Ca^2+^-buffering and Ca^2+^-sensing abilities of CASQ1 are not two different roles but are functionally and structurally correlated to each other. In short, the Ca^2+^-dependent dynamic polymerization and depolymerization of CASQ1 provide the SR with a rapidly releasable Ca^2+^ reservoir during skeletal muscle contraction and a highly negatively charged surface that easily absorbs Ca^2+^ at rest or during relaxation. The differences in their abilities in front-to-front and back-to-back interactions and in the composition of the asp-rich/CAS region in CASQ1 and CASQ2 generate isoform-specific Ca^2+^-binding capacities and different Ca^2+^-dependent polymerization properties^51,73,82^.

Ca^2+^ buffering by CASQ1 in the SR during Ca^2+^ release from the SR shows a nonlinear pattern^51,83,84^, which is explained by the polymerization-dependent Ca^2+^-binding capacity of CASQ1. In the SR fully loaded with Ca^2+^, CASQ1 is polymerized and bears many Ca^2+^ to prepare for Ca^2+^ release from the SR via RyR1. At the beginning of Ca^2+^ release from the SR, [Ca^2+^] at the release site of the SR decreases, which causes the depolymerization of CASQ1 at the site and subsequent release of Ca^2+^ from the depolymerized CASQ1 to the SR lumen via the loss of Ca^2+^-binding ability (unbuffering of Ca^2+^ by CASQ1). More Ca^2+^ release from the SR to the cytosol can occur by the unbuffering of Ca^2+^ by depolymerized CASQ1. A further cycle of depolymerization and Ca^2+^ unbuffering of CASQ1 results in more rapid decay of [Ca^2+^]_SR_ compared with the beginning of Ca^2+^ release (i.e., a nonlinear pattern). Concurrently, the depolymerized CASQ1 detaches from the JSR membrane, which induces the loss of inhibitory action of CASQ1 on RyR1 activity^72,85–87^. Further depolymerization of CASQ1 finally terminates Ca^2+^ release from the SR through RyR1 (i.e., inactivation of RyR1) when Ca^2+^ depletion from the SR and the detachment of CASQ1 from the JSR membrane progress further^74,75^. In addition, the binding of CASQ1 to RyR1 is also eliminated by increasing [Ca^2+^]_SR_ to ≥4 mM (probably mimicking the state of the SR at rest), which consequently eliminates the inhibitory effect of CASQ1 on RyR1 activity^54,67,88^.

## Regulation of RyR1 activity by a quaternary complex of CASQ1/RyR1/triadin/junctin

The acidic asp‐rich/CAS region of CASQ1 in the C-terminus directly interacts with triadin via a KEKE motif in triadin (localized in the SR lumen), which helps CASQ1 anchor to the JSR membrane and allows CASQ1 to bind to Ca^2+^ in the area near RyR1^89–91^. The same luminal KEKE motif of triadin also interacts with the C-terminal loop of RyR1^89,92,93^. Triadin physically links CASQ1 to RyR1, forming a bridge between CASQ1 and RyR1^89^. It has also been reported that CASQ1 directly interacts with RyR1^94^. The acidic asp‐rich/CAS region of CASQ1 also interacts with junctin via the KEKE motif^95,96^. All these reports suggest that CASQ1 can participate in generating a protein complex with RyR1, junctin and triadin (i.e., a quaternary complex of CASQ1/RyR1/triadin/junctin).

The possibility has been suggested that the N-terminal residues of CASQ1 may also be responsible for its binding to triadin and junctin, and the CASQ1 polymer may not bind to these proteins^53^. However, according to EM observations of skeletal muscle and other studies on the binding site of CASQ1 to triadin, as mentioned above^61,90,97^, the CASQ1 polymer can form a complex with RyR/triadin/junctin. At 1 mM [Ca^2+^]_SR_, the CASQ polymer is stable and anchors to the JSR membrane through triadin and junctin, and further increasing [Ca^2+^]_SR_ to over 10 mM results in the dissociation of CASQ from triadin and junctin^50,67,98^, which is in accordance with the observation that the CASQ1 polymer at ≥4 mM [Ca^2+^]_SR_ causes a gradual dissociation of CASQ1 from junctin, triadin and RyR1^54,67,69,88^. Interestingly, both the formation of the quaternary complex and the formation of CASQ1 polymers contribute to the retention of CASQ1 inside the SR in skeletal muscle^99^.

CASQ1, triadin, and junctin are not essential for survival or skeletal muscle contraction; however, they are related to RyR1 activity and the gain of EC coupling in skeletal muscle. As mentioned in the previous section of this review, CASQ1 acts as a Ca^2+^ sensor to modulate the activity of RyR1. In early studies, the functional relevance of the interaction between CASQ1 and RyR1 was controversial. CASQ1 was originally described as an activator of RyR1 in isolated heavy SR vesicles^100^. In a single-channel study with a planar lipid bilayer, the addition of purified CASQ1 to the luminal side enhanced the activity of purified RyR1^101^. Overexpression of CASQ1 in C2C12 skeletal myotubes enhanced caffeine- and membrane depolarization-induced intracellular Ca^2+^ release through RyR1^102^. Overexpression of a deletion mutant of CASQ1 lacking the C-terminal aspartate-rich/CAS region (the triadin- or junctin-binding domain) in C2C12 myotubes resulted in reduced caffeine- and membrane depolarization-induced Ca^2+^ release^90,102^. Even a case of no functional relationship between CASQ1 and RyR1 was reported (i.e., no significant functional effect of CASQ1 on RyR1)^103^. Other groups reported that CASQ1 alone could activate RyR1 activity^104^, but CASQ1 inhibited RyR1 activity by binding to triadin^67,104^. Deletion of the C-terminal acidic asp‐rich/CAS region of CASQ1 abolished the inhibitory action of CASQ1 on RyR1^96^. Specifically, in a single-channel study with a planar lipid bilayer, the addition of purified triadin or junctin to the luminal side increased the activity of purified RyR1, and the addition of purified CASQ1 abolished the effect of junctin on RyR1 but not the effect of triadin^105^. The different compositions of RyR or CASQ isoforms, the different degrees of expression levels of RyR1 or CASQ1 (or the ratio between them), and the different salt concentrations in the various experimental conditions could cause discrepancies in the role of CASQ1 in RyR1 activity. For example, CASQ1 was dissociated from the JSR membrane by increasing the luminal ionic strength (under high-salt conditions), and the inhibitory effect of CASQ1 on RyR1 activity was removed by the dissociation of CASQ1^67^. After all of these reports, it has finally been accepted that at low [Ca^2+^]_SR_, CASQ1 functions as a Ca^2+^ sensor that inhibits RyR1 activity in the presence of triadin at least (considering the quaternary complex, in the presence of both triadin and junctin) to preserve a certain level of Ca^2+^ in the SR^50,51,53,67,72,94^. Consistently, triadin knockout mice show skeletal muscle weakness with reduced expression of CASQ1^106^, and a human patient with a triadin mutation also shows skeletal muscle weakness^107^. For more details on RyR1 activity along with its partners or regulatory proteins under various pathological conditions, including MH, skeletal muscle myopathies, cardiac arrhythmias, epilepsy, neurodegeneration, and pain, please refer to a review article^108^.

Overall, the regulation of RyR1 activity by CASQ1 is not a simple one-way process but a very complicated and bidirectional process that is optimized for the fine-tuning of skeletal muscle contraction or relaxation, with dependencies on [Ca^2+^]_SR_, the phosphorylation status of CASQ1 (see the next section in this review), the polymerization status of CASQ1, binding to other proteins such as triadin and junctin (i.e., the quaternary complex of CASQ1/RyR1/triadin/junctin), and even RyR1 activity itself (i.e., the nonlinear relationship between Ca^2+^-buffering and Ca^2+^ release from the SR, as discussed before).

## Phosphorylation of CASQ1

The phosphorylation status of CASQ1 also affects the Ca^2+^-binding capacity of CASQ1 by regulating the interaction of CASQ1 with other proteins. CKII (which can phosphorylate serine or threonine residues) is present in the SR lumen in rabbit fast-twitch skeletal muscle^109^. The threonine located at the beginning of the C-terminus of CASQ1 is the only site of CASQ1 phosphorylated by CKII (at 353 in the acidic region, I^351^NTEDDDDDE-COOH)^55^. The acidic region partly overlaps with the triadin-binding region (residues 354-367^90^) and lies near the electronegative pocket formed by the front-to-front interaction during CASQ1 polymerization^53^. Phosphorylation of CASQ1 at the threonine residue increases the Ca^2+^-binding capacity of CASQ1 nearly 2-fold^110^.

Isolated CASQ1 exists in both phosphorylated^88,111^ and dephosphorylated forms^55,112^. It is controversial whether the phosphorylation of CASQ1 affects the interactions between CASQ1 and RyR1 or the inhibitory effect of CASQ1 on RyR1. Only dephosphorylated CASQ1 can enhance RyR1 activity without binding to triadin and junctin (approximately fivefold increase in open probability and approximately twofold increase in mean open time at 1 mM of [Ca^2+^]_SR_), and only 1% dephosphorylated CASQ1 in the total CASQ1 is sufficient to enhance RyR1 activity^113^. However, the affinity of phosphorylated CASQ1 to RyR1 is similar to that of dephosphorylated CASQ1^94^. In contrast, both phosphorylated and dephosphorylated CASQ1 have an inhibitory effect on RyR1 activity at 1 mM [Ca^2+^]_SR_ and only when the quaternary complex of CASQ1/RyR1/triadin/junctin is intact^67,88,110^. The enhancement of RyR1 activity by dephosphorylated CASQ1 is observed only when CASQ1 is bound to triadin alone^110^. Considering that functional RyR1 is a very large ion channel (i.e., tetrameric RyR1s with more than 2 MDa), the ambiguity in the relationship between RyR1 activity and CASQ1 could possibly be due to the participation of other proteins that can bind RyR1 and/or exert regulatory effects on RyR1 activity. In addition, interestingly, CASQ1 itself has kinase activity in the presence of Mg-ATP (which is present in the SR) and can autophosphorylate^111,114^, suggesting that CASQ1 phosphorylates other proteins in the SR, such as EC coupling-mediating proteins, including RyR1. These various reports surely suggest that the regulation of RyR1 activity by CASQ1 depends on the phosphorylation state of CASQ1, [Ca^2+^]_SR_, and the binding of triadin and junctin to CASQ1. Overall, the relationship between the phosphorylation status of CASQ1 and the regulation of RyR1 activity by CASQ1 via interaction between RyR1 and CASQ1 should be further investigated.

The Ca^2+^-binding capacity of dephosphorylated CASQ1 is reduced by more than one-third compared with that of phosphorylated CASQ1^53,110^. Phosphorylation of CASQ1 does not alter CASQ1 polymerization^110^. Therefore, it is likely that phosphorylated CASQ1 corresponds to polymerized CASQ1.

## CASQ1 deficiency

**Fig. 2:**
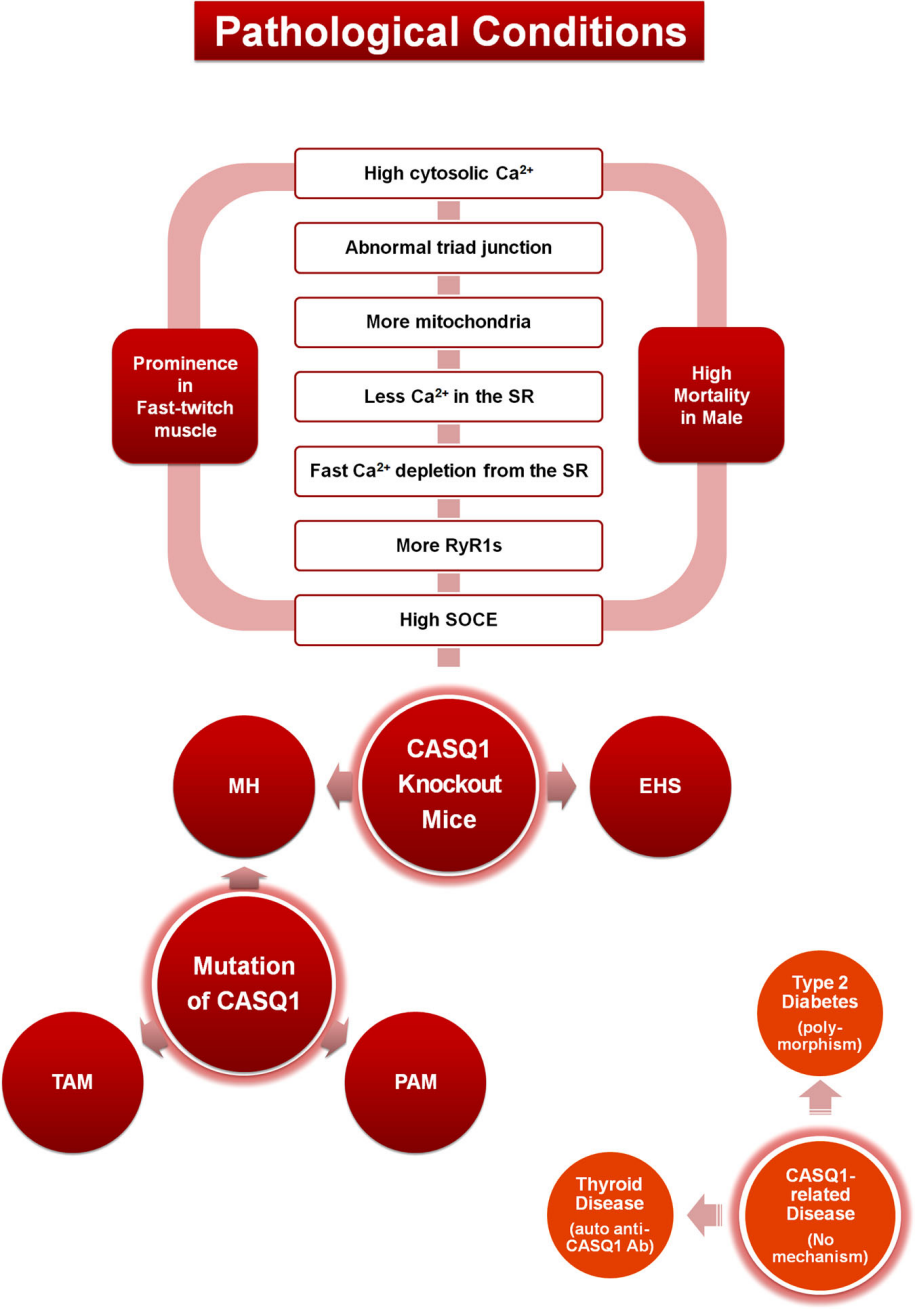
Summary of pathological involvement of CASQ1 in skeletal muscle. Pathological involvement of CASQ1 in skeletal muscle is summarized in circles (red). In the lower right (deep orange circles), diseases that are related to CASQ1 with no known mechanism are summarized. Corresponding proteins, mechanisms or phenomena are presented in parentheses. Pathological phenomena and tendencies due to the knockout of CASQ1 in mice are listed in the boxes. CASQ calsequestrin, RyR1 ryanodine receptor 1, SR sarcoplasmic reticulum, SOCE store-operated Ca^2+^ entry, MH malignant hyperthermia, EHS environmental/exertional heat stroke, TAM tubular aggregate myopathy, PAM protein aggregate myopathy, Ab antibody.

CASQ1 knockout mice are viable, fertile, and have normal muscle performance, although mild atrophy is observed^115^. Skeletal muscle tissues and fibers from CASQ1 knockout mice show remodeling of the triad junction, such as a narrow lumen of the JSR caused by shrinking, the proliferation of triad-like multilayered junctions (that differ from the typical triad junction, in approximately 70% of EDL fibers, and even in the LSR), an increase in the number and volume of mitochondria, and an increase in the number of RyR1 molecules (even in multilayered junctions) (Fig. 2)^115,116^. Therefore, CASQ1 is essential for the normal development of the SR and triad junction (which is the third role of CASQ1 in skeletal muscle, Fig. 1), as well as for Ca^2+^-buffering and Ca^2+^-sensing in the SR to release appropriate amounts of Ca^2+^.

The amount of Ca^2+^ in the SR of fast-twitch EDL skeletal muscle fibers from CASQ1 knockout mice is reduced compared with that in wild-type fibers^65,115^. The appearance of multilayered junctions with RyR1s could be a compensatory response to the reduced SR Ca^2+^ storage with the shrinkage of terminal cisternae. There is no compensatory increase in the expression of CASQ2 in either fast- or slow-twitch skeletal muscles^115^. Fast-twitch EDL skeletal muscle fibers from CASQ1 knockout mice display similar free [Ca^2+^]_cytosol_ but significantly reduced Ca^2+^ release from the SR in response to electrical stimulation or caffeine compared with wild-type fibers, while slow-twitch soleus skeletal muscle fibers are not affected^115,117^. However, tension developed during isometric tetanus for a short duration is not significantly different in the two types of muscle fibers, and the sensitivity to caffeine is also unchanged (similar EC_50_ values)^115,116^. Fast-twitch EDL skeletal muscle fibers from CASQ1 knockout show prolonged twitch time parameters (time-to-peak or half-relaxation time, i.e., delayed Ca^2+^ release or removal)^115^, which conflicts with the reduced Ca^2+^ release from the SR in response to electrical stimulation or caffeine in the same EDL fibers. These unexpected results can be induced by an impaired ability of Ca^2+^ reuptake to the SR or a loss of CASQ1-mediated inhibitory effect on RyR1 activity during EC coupling. The increased number of mitochondria is related to the higher resistance of CASQ1 knockout mice to fatigue than wild-type mice^115^. EDL muscles from CASQ1 knockout mice also show mitochondrial damage with oxidative stress^116,118^.

There are no marked differences between mice carrying CASQ1 single- and CASQ1/CASQ2 double-knockouts^115,116,119^. Fast-twitch FDB skeletal muscle fibers from single- or double-knockout mice do not sustain prolonged muscle activity (such as repetitive electrical stimuli) due to faster SR Ca^2+^ depletion, a decrease in [Ca^2+^]_SR_, impaired Ca^2+^ reuptake to the SR and finally an inability to sustain [Ca^2+^]_cytosol_ during contraction^119^. The proper function of fast-twitch FDB skeletal muscle fibers from double-knockout mice requires external Ca^2+^ entry (i.e., SOCE)^119^, suggesting a possible relationship between CASQ1 and SOCE (see the next part of this review). Unlike CASQ1 single-knockout mice, CASQ1/CASQ2 double-knockout mice show a more severely damaged structure of the soleus muscle (alterations in ~30% of soleus fibers but not in EDL fibers), although both single- and double-knockout mice exhibit significant reductions in body weight and grip strength^116^. However, under tetanic stimulation (2 s and 100 Hz), the soleus was able to sustain contraction, while active tension in the EDL declined by 70–80%. This discordance in the structural damage and function in the slow-twitch soleus skeletal muscle fibers is explained by the nature of the skeletal muscle type: fast-twitch EDL skeletal muscle fibers need the release of large amounts of Ca^2+^ from the SR during tetanic stimulation; in contrast, slow-twitch soleus fibers require less Ca^2+^ and have slower cycling than fast-twitch EDL skeletal muscle fibers.

Transient knockdown of CASQ2 alone or both CASQ1 and CASQ2 in C2C12 myotubes induces a reduction in the SR Ca^2+^ content due to the reduced expression and lower activity of SERCA along with a reduction in the expression of RyR1 compared to wild-type myotubes^101^.

## A reverse-directional signal from CASQ1 to SOCE

In most cell types, the Ca^2+^ gradient between the cytosol (approximately 100 nM) and extracellular space (mM range) is maintained (approximately 20,000-fold), which suggests that extracellular Ca^2+^ is an effective source of intracellular Ca^2+^ signals^120^. SOCE is one extracellular Ca^2+^ entry route in various cells^121^. Generally, SOCE is operated by coordinated interactions between STIMs (STIM1, STIM1L, and STIM2) and Ca^2+^ release-activated Ca^2+^ channel proteins (Orai1, Orai2, and Orai3). STIM1 and Orai1 are the major isoforms mediating SOCE. STIM1 in the ER membrane senses the depletion of Ca^2+^ from the ER via its EF-hand motif, and it activates Orai1 in the plasma membrane via a physical interaction between the cytoplasmic C-terminus of STIM1 and the STIM-Orai1-activating region (SOAR) of Orai1. During this process, sensing Ca^2+^ by STIM1 (i.e., Ca^2+^ depletion from the ER) induces the homo-oligomerization of STIM1 and its relocalization to the ER membrane near the plasma membrane where Oria1 is located, which also induces the homo-oligomerization of Orai1. The final hetero-oligomers that are formed by STIM1s and Orai1s are called ‘puncta’.

The presence of SOCE in skeletal muscle was first identified in 2001^122^. There are several differences between SOCE in skeletal muscle cells and general SOCE in another cell types^5–7^. First, the SR or t-tubule in skeletal muscle corresponds to the ER or plasma membrane, respectively. Second, without Ca^2+^ depletion from the SR, puncta are formed during the development of skeletal muscle fibers and the process of terminal differentiation after birth (i.e., prepuncta formation)^13,123,124^. However, the prepuncta are not functional, and the additional conformational changes in Orai1 and/or STIM1 within the prepuncta are required to evoke SOCE^13,123^. An advantage of prepuncta formation in skeletal muscle is faster kinetics (rapid Ca^2+^ entry into the cytosol during skeletal SOCE, in less than 1 s, which is significantly faster than in other types of cells (several seconds)^124–126^. In skeletal muscle fibers, SOCE is necessary to restore the SR with Ca^2+^ to the resting level and to sustain Ca^2+^ in the cytosol for skeletal muscle contraction during tetanic stimulation or fatigue^57,124,125,127,128^. In addition, it has been found that skeletal SOCE requires both structural and functional interactions with other proteins, such as MG29 (which is required for the formation of the triad junction, mentioned before in this review), RyR1, and RyR3 in the triad junction^129^.

The fourth role of CASQ1 in skeletal muscle is as a regulator of SOCE via physical interaction between STIM1 and the acidic asp-rich/CAS region of CASQ1 (Fig. 1)^102,130,131^. Overexpression of CASQ1 in C2C12 myotubes inhibits SOCE^102^, while SOCE is increased in FDB skeletal muscle fibers from CASQ1 single- or CASQ1/CASQ2 double-knockout mice^130,132^. Therefore, CASQ1 not only modulates intracellular Ca^2+^ release but also provides a “reverse-directional signal” to regulate SOCE in skeletal muscle (a signal in the opposite direction of the orthograde signal from DHPR in the t-tubule membrane to RyR1 in the SR membrane during EC coupling, i.e., a signal from CASQ1 in the SR to Orai1 in the t-tubule/plasma membrane)^102,133^, suggesting that the functional interplay among CASQ1, RyR1 and SOCE could also contribute to pathological Ca^2+^ overload as well as to physiological Ca^2+^ signals. Indeed, SOCE is involved in muscle pathophysiology (see the next section on CASQ1-mediated diseases in this review)^129,134^.

The increased SOCE in FDB skeletal muscle fibers from CASQ1 single- or CASQ1/CASQ2 double-knockout mice^130,132^ could be one explanation for the sustained high resting [Ca^2+^]_cytosol_ under reduced Ca^2+^ in the SR and the increased rate of Ca^2+^ depletion from the SR in knockout fibers from these mice^65,67,116,119,135^.

## Other CASQ1-binding proteins

Junctional protein 45 (JP-45, a single transmembrane protein) interacts with two EC coupling-mediating proteins: the α1S subunit of DHPR via its cytosolic N-terminus and CASQ1 via its luminal C-terminus^136,137^. Knockout of JP-45 in young mice results in a loss of skeletal muscle strength due to decreases in the expression and charge movement of DHPR and in membrane depolarization-induced internal Ca^2+^ release through RyR1 in FDB skeletal muscle fibers^138^. However, it is controversial whether JP-45 affects the activity of DHPR because both overexpression and knockdown of JP-45 in C2C12 myotubes result in a decrease in the charge movement of DHPR^137^. Surprisingly, the skeletal muscle phenotype of young JP-45 knockout mice is similar to that of aged mice: reductions in the expression of DHPR and JP-45 and in intracellular Ca^2+^ release in response to membrane depolarization likewise occur in aged mice^139,140^.

Ca^2+^ entry through DHPR during skeletal EC coupling (so-called ECCE) is crucial to maintain muscle force development in the mouse slow-twitch soleus muscle, and ECCE is increased in FDB skeletal muscle fibers from JP-45/CASQ1 double-knockout, JP-45/CASQ2 double-knockout or JP-45/CASQ1/CASQ2 triple-knockout mice^141^, suggesting another type of ‘reverse-directional signal’ by CASQ1 via complex formation with JP-45. However, it is also controversial whether JP-45 affects ECCE through DHPR. Overexpression of JP-45 in C2C12 myotubes does not induce much change in the voltage-activated inward Ca^2+^ current (corresponding to ECCE in skeletal muscle fibers or myotubes)^142^. Therefore, more dedicated examinations are required to clarify the effects of JP-45 on DHPR or ECCE through DHPR in skeletal muscle.

CASQ1 prevents the dimerization of inositol-requiring enzyme 1α (IRE1α, a sensor of ER stress) by interacting with the luminal domain of IRE1α at JSR, which attenuates IRE1α signaling at physiological Ca^2+^ levels such as during skeletal muscle contraction or relaxation^143^. In cardiac muscle, IRE1a signaling plays protective roles under cardiac fibrosis or atherosclerosis^144,145^. These reports suggest the possibility that CASQ1 could participate in the stress-coping responses in skeletal muscle as well as cardiac muscle.

## Other Ca^2+^-buffering proteins in the SR and CASQ-like proteins

Other Ca^2+^-buffering proteins in the SR have been reported, such as histidine-rich Ca^2+^-binding protein (HRC, 160 kDa) and sarcalumenin (SLM, 150 kDa)^109,146,147^. The amounts of HRC and SLM in SR are much lower than the number of CASQ1^109^. Similar to CASQ1, HRC, and SLM are acidic and act as substrates of CKII. Unlike CASQ1, which is mainly located in the terminal cisternae of the SR, HRC, and SLM are more widely distributed and buffer Ca^2+^ throughout the SR^148^. The Ca^2+^-dependent phosphorylation of SLM and HRC by CKII inhibits the binding affinity of RyR1 to Ca^2+^^147,149^. This report suggests that HRC and SLM could regulate RyR1 in a Ca^2+^- or phosphorylation-dependent manner. In addition to CKII, HRC can be phosphorylated by Ca^2+^/calmodulin-dependent protein kinase II^146^. A 53-kDa Ca^2+^-binding protein is a splice variant of SLM^150^.

Calreticulin, also known as calregulin, is the ER homolog of CASQ1, and it also exists in skeletal muscle^151,152^. Calreticulin has no EF-hand consensus sequence; however, the highly acidic C-terminus of calreticulin accounts for its low-affinity and high-capacity Ca^2+^ binding properties.

CASQ-like proteins have been found in other organisms or tissues, including in smooth muscles of the rat aorta and stomach^48^, in the urinary bladder of guinea pig^153^, in Purkinje cells of chicken cerebellum^154^ and in the liver of rat^155^, but their functions have not been studied. A CASQ-like protein is found in body wall muscle cells of *Caenorhabditis elegans* (50% similarity and 30% identity to rabbit CASQ1), but it is not essential for body wall muscle formation and contraction^156^. CASQ-like proteins that are recognizable by anti-CASQ1 antibodies are found in the eggs of sea urchins (*Strongylocentrotus droebachiensis* and *Arbacia punctulata*)^157^.

Interestingly, CASQ-like proteins have also been found in plants, such as cultured cells of *Streptanthus tortuosus* or spinach leaves^158^. The CASQ-like proteins in plants can bind to Ca^2+^ and are recognized by the anti-CASQ2 antibody. The other plant CASQ-like protein is found in Ca^2+^-accumulating cells from water lettuce (*Pistia stratiotes*)^159^. Various CASQ-like proteins in various types of cells and tissues from diverse species strongly suggest that the functions of CASQ1 could be more diverse than previously known.

## CASQ1-related diseases

Tubular aggregate myopathy (TAM) was originally associated with mutations in STIM1 or Orai1, along with the dysregulation of SOCE (higher or lower SOCE than normal)^160–162^. Patients with heterozygous missense mutations in the *CASQ1* gene (D44N, N56Y, G103D, and I385T) show TAM^163,164^. I385T shows a slight increase in Ca^2+^-dependent tubular aggregation, but D44N, N56Y, and G103D show a decrease in Ca^2+^-dependent tubular aggregation. Interestingly, all the mutants reduced the amount of Ca^2+^ in the SR and D44N and I385T additionally showed a defect in the ability to inhibit SOCE^163^, again confirming that CASQ1 participates in the modulation of SOCE-mediated Ca^2+^ homeostasis via a reverse-directional signal.

D244G (a heterozygous missense mutation in the *CASQ1* gene) was the first identified CASQ1 mutant in patients showing mild myopathy characterized by muscle weakness, fatigue, and the presence of large vacuoles^165–167^. D244G induces misfolding and abnormal aggregation of CASQ1 (which is different from the physiological CASQ1 polymers) due to the loss of the electric charge in D244 that is involved in the Ca^2+^-binding and Ca^2+^-dependent polymerization of CASQ1. Abnormal CASQ1 aggregates cause sarcoplasmic vacuolar aggregations (which induce protein aggregate myopathy (PAM)) and induce reduced Ca^2+^ release from the SR.

Under the administration of volatile anesthetics such as isoflurane, sevoflurane, halothane, desflurane, and enflurane or muscle relaxants such as succinylcholine, patients with MH (a pharmacogenetic skeletal muscle disorder) can undergo sudden death by acute life-threatening skeletal muscle contracture with hypermetabolism that is characterized by increased O_2_ consumption, an extreme elevation in body temperature and rhabdomyolysis^168–170^. The main cause of the crisis in MH patients is the uncontrolled elevation of [Ca^2+^]_cytosol_ that is mediated by uncontrolled activation of dominant mutants of RyR1^168,169^. Hundreds of mutations in the *RyR1* gene have been found in MH patients^171^, and most of these mutations are confined to three regions (called hot spots)^172^: C35-R614 in the N-terminus, D2129-R2458 in the central region, and I3916-G4942 in the C-terminus. MH is also attributed to mutations in the *CACNA1S* gene encoding the α1S subunit of DHPR^173^. In addition to the mutations in RyR1 and DHPR, sustained SOCE may affect the intracellular Ca^2+^ balance in the skeletal muscles of MH patients^174^. Until now, the only clinical treatment of MH has been the administration of dantrolene (a muscle relaxant)^1,175–178^. However, the molecular mechanism of the dantrolene effect has not been clearly demonstrated. What is the direct target of dantrolene? What proteins or pathways are involved in the dantrolene effect?

**Fig. 3:**
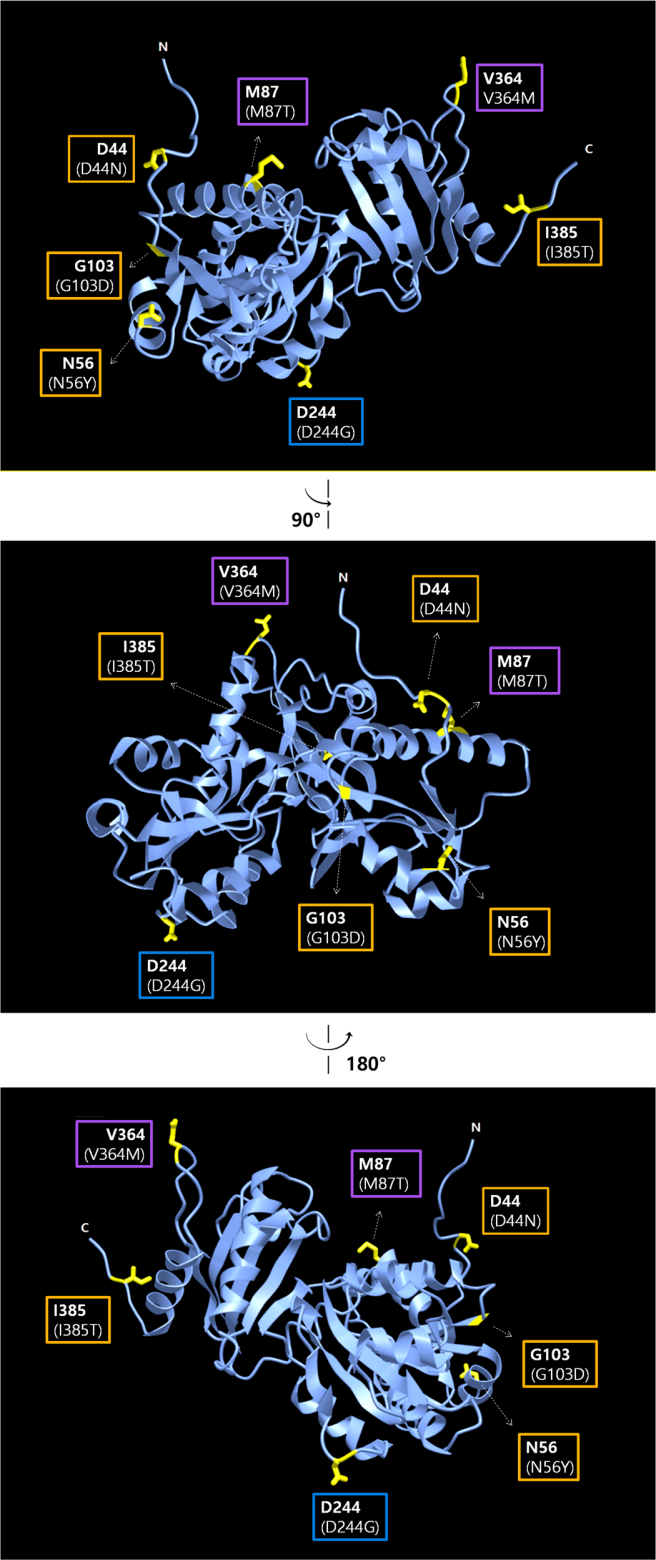
3D structures of human CASQ1 with indications of residues that are related to human skeletal muscle diseases. The 3D structure of human CASQ1 (PDB ID: 3UOM) is presented in the top panel as a ribbon diagram using iCn3D (NCBI’s web-based 3D structure viewer^202^, https://www.ncbi.nlm.nih.gov/Structure/mmdb/mmdbsrv.cgi?Dopt=s&uid=97449). The top image is rotated around the vertical axis (90° or 180° counterclockwise) and presented in the middle or bottom image, respectively. Residues that are involved in human skeletal muscle diseases are presented in the boxes and highlighted in yellow in the 3D structures, and their side chains are presented as sticks. Mutations that are involved in human skeletal muscle diseases are presented in parentheses in the boxes. Numbers indicate the amino acid sequence positions. N or C indicates the N- or C-terminus, respectively. The signal peptide in the N-terminus is not shown. The residues or mutations in the orange boxes are related to TAM (tubular aggregate myopathy); the residues or mutations in the purple boxes are related to MH (malignant hyperthermia); the residue or mutation in the blue box is related to PAM (protein aggregate myopathy).

Interestingly, mutations in CASQ1 in human patients or knockouts of CASQ1 in mice induce symptoms similar to those observed in human patients with MH. Patients with M87T (a missense mutation in the *CASQ1* gene) show a mild association with MH^167^. M87T induces a mild reduction in the Ca^2+^-binding capacity of CASQ1 due to the shift of the M87-bearing α-helix and the weakening of the back-to-back interaction that is essential for the dimerization of CASQ1. CASQ1 knockout mice clearly show MH-like gain-of-function phenotypes that are very similar to those of RyR1 knock-in mice expressing human MH mutations (Y522S or R163S, gain-of-function mutations in RyR1)^135,179–183^, such as impaired movement, difficulty breathing, whole-body contractions, an arched back and finally rapid progression to sudden death. Loss of the inhibitory effect of CASQ1 on RyR1 activity and Ca^2+^ depletion from the SR due to the absence of CASQ1 explain the MH-like phenotypes in CASQ1 knockout mice^67,119^. A mutation in the *CASQ1* gene (1090 G > A in the nucleotide sequence; V364M in the amino acid sequence) has been reported in MH patients, although its pathological mechanism has not yet been studied^184^. The locations of mutations that are related to patients with MH, TAM, or PAM are indicated in the 3D structure of human CASQ1 (Fig. 3).

Unexpectedly, patients with environmental/exertional heat stroke (EHS, triggered either by challenging environmental conditions (such as hot temperatures and high humidity) and/or strenuous exercise) exhibit remarkably similar symptoms to those in patients with MH^185^, such as lethal hypermetabolic crisis with increases in ATP demand, in body temperature, in contractile sensitivity to temperature, caffeine, and membrane depolarization, and in resting Ca^2+^, rhabdomyolysis and finally sudden death in response to heat exposure or physical exertion. These EHS symptoms are also found in CASQ1 knockout mice. For example, at low temperature (25 °C), resting free [Ca^2+^]_cytosol_ and [Ca^2+^]_SR_ is not significantly different between fast-twitch FDB skeletal muscle fibers from CASQ1 knockout and wild-type mice^119^. However, at >30 °C, [Ca^2+^]_cytosol_ is significantly increased in FDB skeletal muscle fibers from CASQ1 knockout mice^135^. In addition, slow-twitch soleus muscle fibers from CASQ1 knockout mice are not significantly affected by temperature challenge^135^. These results from CASQ1 knockout mice suggest that the development of increased basal tension in fast-twitch muscle and the impaired control of [Ca^2+^]_cytosol_ could be the starting point toward the development of contractures during MH or EHS crises (Fig. 2). Therefore, the *CASQ1* gene could be a powerful candidate for examining MH and EHS patients who do not have a mutation in the *RyR1* or *DHPR* gene.

Here is “a possible scenario” for the MH- or EHS-like phenomena in CASQ1 single- or CASQ1/CASQ2 double-knockout mice^65,67,115,116,119,130,132,135,172,186–188^. First, structural remodeling of the triad junction, such as shrinking and abnormal multilayered triad junctions, occurs. Second, CASQ1 knockout induces the loss of inhibitory action of CASQ1 on RyR1 activity. Third, the absence of CASQ1 induces more rapid Ca^2+^ depletion from the SR due to the absence of Ca^2+^-buffering ability by CASQ1. Fourth, when exposed to halothane or heat, the knockout fibers show insufficient Ca^2+^ from the SR due to faster Ca^2+^ depletion from the SR with impairment of Ca^2+^ reuptake through SERCA1a into the SR. Fifth, more rapid Ca^2+^ depletion from the SR lowers the threshold for store overload-induced Ca^2+^ release (SOICR^188^). Sixth, the rapid Ca^2+^ depletion from the SR also increases skeletal SOCE to refill the SR. Seventh, the impaired SERCA1a activity probably increases ATP consumption, thus requiring a greater number of mitochondria (i.e., a hypermetabolic state occurs along with increased metabolic wastes and ionic disturbance^115^). Eighth, all these abnormal events induce more increases in cytosolic [Ca^2+^]. Correlations between the abnormal events mentioned above and the causative roles of CASQ1 in MH in humans should be further investigated, although it has been revealed that each abnormal event is related to the characteristic muscle rigidity and rhabdomyolysism showed in patients with MH or EHS and death.

An unexpected property has been observed in CASQ1 knockout mice: a sex dependency. When CASQ1 knockout mice are exposed to halothane (2%, 1 h) or to high environmental temperature (41 °C, 30 min), male CASQ1 knockout mice exhibit a high mortality rate (80%, particularly after 3 months), but female CASQ1 knockout mice show only marginal effects^135^. This sex-dependent mortality rate has also been found in human patients with MH or EHS^189,190^. The mechanisms responsible for the higher mortality rate in males have not been studied. A possible explanation for the sex-dependent mortality rate could be hormonal differences between males and females (related to different muscle masses and capacities).

Although the reduction or deficiency of dystrophin is the primary pathological cause of Duchenne muscular dystrophy (DMD, a lethal form of skeletal muscular dystrophy characterized by progressive wasting of the skeletal muscle^191^), dystrophic skeletal muscle fibers from a murine animal model of DMD (i.e., *mdx* mice) show reduced expression of CASQ-like proteins of 150–220 kDa (with no change in the expression level of RyR1, DHPR, SERCA1a, and CASQ1)^192^. The decreased expression of CASQ-like proteins might be related to the altered Ca^2+^ level in the SR and cytosol and, subsequently, muscle necrosis or apoptosis in DMD patients. In addition, skeletal muscle fibers from another *mdx* mouse show excessive SOCE due to the increased expression of Orai1^193^, as shown in FDB muscle fibers from CASQ1 single- or CASQ1/CASQ2 double-knockout mice^130,132^. These findings suggest a strong possibility that, in addition to the main pathological role of dystrophin in DMD (i.e., muscle damage due to the reduction or deficiency of dystrophin by mutations), abnormal Ca^2+^-buffering by CASQ1 and/or CASQ-like proteins in the SR, in combination with excessive SOCE, could be related to the pathological mechanism of DMD.

Several other pathological conditions are related to CASQ1, although clear correlations between the pathological phenotypes of patients and CASQ1 have not been defined. CASQ1 is related to diabetes. A diabetic rat model has shown increased expression of CASQ1 with significantly increased Ca^2+^-buffering capacity^194^. Polymorphisms in the *CASQ1* gene are found in the Caucasian population in North America and in the Amish population with type 2 diabetes, suggesting that sequence variation in the *CASQ1* gene could increase the risk for type 2 diabetes^195,196^. CASQ1 is also related to an abnormal thyroid status. Patients with Graves’ disease (a thyroid disease) show increased mRNA levels of both the *CASQ1* and *CASQ2* genes in thyroid tissue^197^. Unlike normal subjects, patients with Graves’ hyperthyroidism without ophthalmopathy and Hashimoto’s thyroiditis with or without ophthalmopathy show significant levels of autoantibodies against CASQ1 in the serum^198,199^. The acute induction of hyperthyroidism in the rabbit soleus muscle increases the mRNA level of CASQ1^200^.

Although not discussed in this review, CASQ2 is closely related to human diseases, especially catecholaminergic polymorphic ventricular tachycardia (CPVT, an arrhythmogenic disorder characterized by physical or emotional stress-induced syncopal events and finally sudden cardiac death). Regarding details on the relationship among CASQ2, RyR2, and CPVT in cardiac muscle, please refer to a review article^201^.

## Conclusion and future directions

For the previous three decades, extensive and dedicated studies on CASQ1 in skeletal muscle have been conducted, and the critical roles of CASQ1 have been revealed, which has been a monumental achievement in our understanding of the physiological and path-physiological properties of skeletal muscle. However, considering that CASQ1 is the major Ca^2+^-buffering protein in the SR of skeletal muscle, it is plausible that the knockout of CASQ1 in skeletal muscle can induce enormous reductions in Ca^2+^ release from the SR in response to various stimuli and in the amount of Ca^2+^ available for release from the SR; however, this is not observed (still significant but only relative reductions). In addition, only MH- or EHS-like symptoms, but not other skeletal muscle disease-like symptoms, have been found in CASQ1 single- or CASQ1/CASQ2 double-knockout mice. Muscle fibers from knockout mice still sustain a sufficient degree of Ca^2+^ release and muscle contraction. These curiosities raise the possibility that either CASQ1 has functions that are not currently understood or other proteins could compensate for the absence of CASQ1 alone or of both CASQ1 and CASQ2.

In addition, no marked difference is observed between CASQ1 single- and CASQ1/CASQ2 double-knockout mice, which raises another important question: what is the physiological relevance of CASQ2 “in skeletal muscle”? It is possible that proteins and/or regulatory mechanisms exist, and further investigations of such proteins and/or mechanisms will help to answer these puzzling questions and improve our understanding of the physiological and pathophysiological properties of skeletal muscle.

In our aging society, the number of patients with skeletal muscle diseases is continuing to increase, and improved understanding of the pathology of skeletal muscle is in great demand. A more detailed understanding of the pathological roles of CASQ1 in skeletal muscle, for example, the roles of CASQ1 in skeletal muscle diseases that are combined with other diseases, could be an excellent way to meet the demand.
